# Electroacupuncture ameliorates cerebral ischemia/reperfusion injury by suppressing autophagy *via* the SIRT1-FOXO1 signaling pathway

**DOI:** 10.18632/aging.103420

**Published:** 2020-07-03

**Authors:** Zhi-Gang Mei, Ya-Guang Huang, Zhi-Tao Feng, Ya-Nan Luo, Song-Bai Yang, Li-Peng Du, Kang Jiang, Xiao-Lu Liu, Xian-Yun Fu, Yi-Hui Deng, Hua-Jun Zhou

**Affiliations:** 1 Key Laboratory of Hunan Province for Integrated Traditional Chinese and Western Medicine on Prevention and Treatment of Cardio-Cerebral Diseases, Hunan University of Chinese Medicine, Changsha, Hunan, China; 2 Third-Grade Pharmacological Laboratory on Chinese Medicine Approved by State Administration of Traditional Chinese Medicine, Medical College of China Three Gorges University, Yichang, Hubei, China; 3 Affiliated Renhe Hospital of China Three Gorges University, Yichang, Hubei, China; 4 Yichang Hospital of Traditional Chinese Medicine, Clinical Medical College of Traditional Chinese Medicine, China Three Gorges University, Yichang, Hubei, China; 5 The Institute of Neurology, The First College of Clinical Medical Sciences, China Three Gorges University, Yichang, Hubei, China

**Keywords:** electroacupuncture, cerebral ischemia/reperfusion, autophagy, SIRT1, FOXO1

## Abstract

Cerebral ischemia/reperfusion (CIR) injury occurs when blood flow is restored in the brain, causing secondary damage to the ischemic tissues. Previous studies have shown that electroacupuncture (EA) treatment contributes to brain protection against CIR injury through modulating autophagy. Studies indicated that SIRT1-FOXO1 plays a crucial role in regulating autophagy. Here we investigated the mechanisms underlying the neuroprotective effect of EA and its role in modulating autophagy *via* the SIRT1-FOXO1 signaling pathway in rats with CIR injury. EA pretreatment at “*Baihui*”, “*Quchi*” and “*Zusanli*” acupoints (2/15Hz, 1mA, 30 min/day) was performed for 5 days before the rats were subjected to middle cerebral artery occlusion, and the results indicated that EA pretreatment substantially reduced the Longa score and infarct volume, increased the dendritic spine density and lessened autophagosomes in the peri-ischemic cortex of rats. Additionally, EA pretreatment also reduced the ratio of LC3-II/LC3-I, the levels of Ac-FOXO1 and Atg7, and the interaction of Ac-FOXO1 and Atg7, but increased the levels of p62, SIRT1, and FOXO1. The above effects were abrogated by the SIRT1 inhibitor EX527. Thus, we presume that EA pretreatment elicits a neuroprotective effect against CIR injury, potentially by suppressing autophagy *via* activating the SIRT1-FOXO1 signaling pathway.

## INTRODUCTION

Stroke, including ischemic stroke and hemorrhagic stroke, is the second largest cause of death globally and the top leading cause of mortality in China, which has the largest stroke burden in the world [1–3]. As the world's largest developing country, China is really facing the challenges of taking care of a rapidly aging population, and the mean age of people with prevalent stroke was 66.4 years [4]. The most common subtype of stroke in China is ischemic stroke, which accounts for 69.6% of all strokes [5]. In addition, the lifetime risk of ischemic stroke is approximately double that of hemorrhagic stroke among both men and women across different regions and sociodemographic index quintiles [6]. Ischemic stroke occurs when there is a sudden occlusion of the arterial blood supply to part of the brain, and it is most commonly manifested by focal neurological deficits. Timely restoration of blood flow and rapid revascularization of the occluded vessels are the most effective approaches to reversing cerebral ischemic damage. Thrombolysis to re-canalize the occlusion and re-perfuse the brain, through either pharmacological or mechanical means are the mainstay treatment options for patients with ischemic stroke, and recombinant tissue-plasminogen activator (r-tPA) is the only drug approved by the U.S. Food and Drug Administration (FDA) for treating ischemic stroke. Delays in achieving reperfusion, incomplete recanalization, hemorrhagic transformation, and secondary injury following reperfusion are some of the limitations of intravenous thrombolysis [7]. Blood reperfusion can result in a series of pathological reactions, including oxidative stress [8], inflammation [9], endoplasmic reticulum stress [10], apoptosis [11, 12], and autophagy [13], leading to cerebral ischemia/reperfusion (CIR) injury, which has been defined as a biochemical cascade causing further worsening of ischemic brain tissue that concomitantly reverses the benefits of restoring circulation following stroke occurrence [14]. Therefore, it is essential to explore an alternative or complementary medicine including numerous types of pretreatment measurements to prevent or limit CIR injury.

Electroacupuncture (EA) is a specialized therapeutic method in which a small electrical charge is applied to needles that are already inserted into specific acupoints and have attained *De Qi* (a composite of unique sensations experienced by patients, commonly described as soreness, numbness, distension, and heaviness; and felt by the acupuncturists as tense, tight, and full) [15]. EA benefits from traditional acupuncture and electrical stimulation, and it can be performed rapidly and is a stable treatment, and the standardized stimulation can be controlled. In addition, EA has been used to treat a broad spectrum of conditions, such as headache, dizziness, facial spasm, depression, anxiety, schizophrenia, Alzheimer's disease, as well as ischemic stroke [16–18]. EA has also been found to be potentially beneficial for CIR injury [19]. Accumulating evidence suggests that EA could dramatically attenuate blood-brain barrier disruption and decrease the volume of cerebral infarction, ameliorate learning and memory impairment as well as neurological deficits in rats with CIR injury [20, 21]. Further studies demonstrated that the beneficial efficacy may be achieved *via* benignly modulating the anti-inflammatory response, oxidative stress, glutamate excitotoxicity, apoptosis, as well as autophagy [22–24].

Autophagy, a highly conserved dynamic process, degrades long-lived or misfolded proteins and damaged organelles, the functions of which decline with advanced aging. Serve as an independent risk factor for chronic disease such as neurodegenerative, cancer and cardiovascular diseases, aging is an irreversible biological process. Studies have shown that autophagy regulates aging-related diseases [25], especially neurodegenerative diseases including Alzheimer's disease [26], Parkinson's disease [27], and ischemic stroke [28]. Autophagy could be exacerbated by ischemia, hypoxia, and stress response during CIR injury [29]. Current studies show that the EA pretreatment could protect brain tissue against CIR injury by suppressing autophagy [24, 30]; however, the mechanisms underlying the efficacy are poorly understood. Silent information regulator 1 (SIRT1) is a NAD-dependent deacetylase that is able to regulate inflammatory responses, oxidative stress, apoptosis, and autophagy by deacetylating histones and non-histones such as the Forkhead box O1 (FOXO1) and p53 [31, 32]. Our previous study indicated that the expression of SIRT1 was significantly downregulated after CIR injury, increasing the expression of SIRT1 could protect brain against CIR injury; while pretreatment with EX527, a SIRT1 inhibitor, could abolish the brain protection of SIRT1 [32]. As the first found transcription factor in the FOXO family, FOXO1 was implicated as a crucial regulator of autophagy in the heart, skeletal muscle, liver, as well as the brain [33]. In the condition of oxidative stress, the expression of acetylated FOXO1 (Ac-FOXO1) increased in the cytosol and interacted with autophagy-related gene 7 (Atg7), one of the essential molecules in autophagy, to activate autophagy [34, 35]. However, further detailed mechanisms have not yet been fully elucidated.

Herein, the present study investigated the mechanism involved in the beneficial effects of EA pretreatment against CIR injury. We hypothesized that EA could ameliorate CIR injury and suppress autophagy during ischemia/reperfusion by activating the SIRT1-FOXO1 signaling pathway.

## RESULTS

### EA pretreatment alleviated neurological deficits and reduced infarct volume in rats with CIR injury

**Figure 1:**
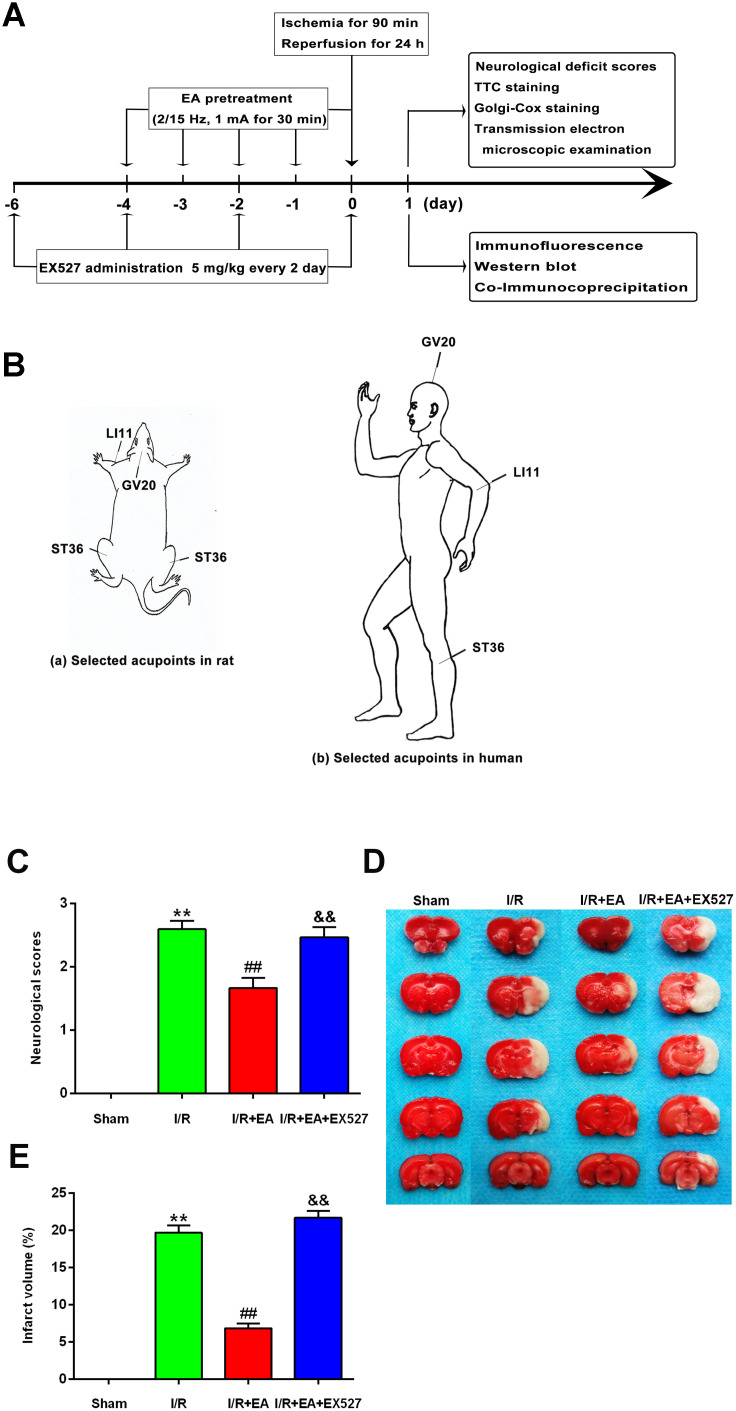
**EA pretreatment ameliorated neurological deficits and reduced infarct volume in rats with CIR injury.** (**A**) The schematic diagram illustrating the chronological events of experiments. (**B**) Rat schematic (a) and human schematic (b) showing the location of the EA acupoints selected in the present study. GV20 stands for “*Baihui*”, which is located at the right midpoint of the parietal bone. LI11 stands for “*Quchi*”, which is located in the depression lateral to the anterior aspect of the radial proximal elbow. ST36 stands for “*Zusanli*”, which is located in the posterolateral side of the knee joint, approximately 5 mm below the capitula fibula. (**C**) Neurological deficit scores (*n*=14). Neurological deficits were evaluated by Longa’s score. (**D**) Images of brain sections by TTC staining. (**E**) The ratio of infarct volume. Data were presented as mean ± SEM (*n*=3). ^**^P<0.01, *vs*. sham group; ^##^*P*<0.01, *vs*. I/R group; ^&&^*P*<0.01, *vs*. I/R + EA group.

To identify the neuroprotective effects of EA at “*Baihui*” (GV20), “*Quchi*” (LI11), and “*Zusanli*” (ST36) acupoints, we first evaluated the neurological deficits and the cerebral infarct volume after 24 h of reperfusion. Rats in the sham group did not exhibit any signs of neurological behavioral dysfunction, whereas rats in the I/R group exhibited significant manifestations of neurological deficits. A great reduction of neurological deficit scores was observed in rats of the I/R + EA group compared with the I/R group. However, I/R + EA + EX527 pretreatment eliminated the decreased neurological scores of the EA pretreatment (Figure 1C).

The infarct volume was measured by TTC staining after 24 h of reperfusion. The white areas represented infarcted brain tissue, and the red areas represented non-infarcted regions (Figure 1D). The infarct volume of rats in the I/R group was markedly larger than that of the sham group. The EA pretreatment significantly reduced the infarct volume compared with the I/R group. However, pretreatment with EA+ EX527 blocked the reduction of cerebral infarction caused by the EA pretreatment (Figure 1E).

### EA pretreatment increased dendritic spine density in cortical neurons of rats with CIR injury

**Figure 2:**
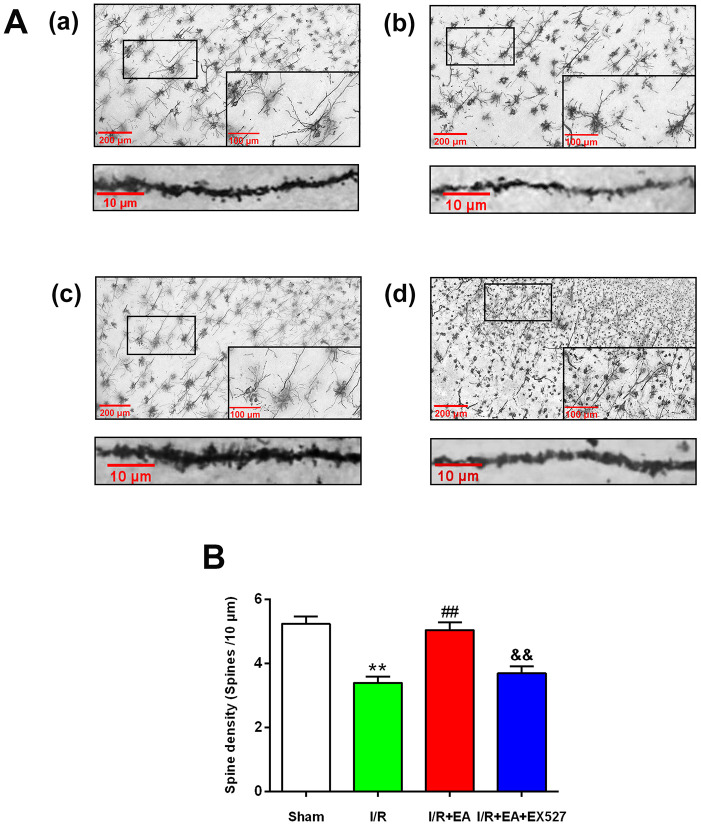
**Effect of EA pretreatment on the dendritic spine density in cortical neurons in rats with CIR injury.** (**A**) The Golgi-cox staining for the dendritic spine of neurons in the peri-ischemic cortex in the sham group (a), the I/R group (b), the I/R + EA group (c), and the I/R + EA + EX527 group (d) (100×). High magnification images are shown in the small windows (400×). The graphs below (a), (b), (c), and (d) were enlarged with 1000×, respectively. Scale bar, 100×: 200 μm, 400×: 100 μm; 1000×: 10 μm. (**B**) The dendritic spine density in cortical neurons. Data were presented as the mean ± SEM (*n*=3). ^**^P<0.01 *vs*. sham group. ^##^*P*<0.01 *vs*. I/R group; ^&&^*P*<0.01 *vs*. I/R + EA group.

We further assessed the effect of EA on the density of dendritic spines in cortical neurons by Golgi-cox staining. The results showed that the dendritic spine density in cortical neurons was markedly decreased in rats of the I/R group compared with the sham group. The EA pretreatment at the GV20, LI11, and ST36 acupoints significantly increased dendritic spine density in cortical neurons. Nevertheless, pretreatment with EA+ EX527 blocked the increase in dendritic spine density improved by the EA pretreatment (Figure 2).

### EA pretreatment decreased the formation of autophagosomes and autophagolysosomes after CIR injury

**Figure 3:**
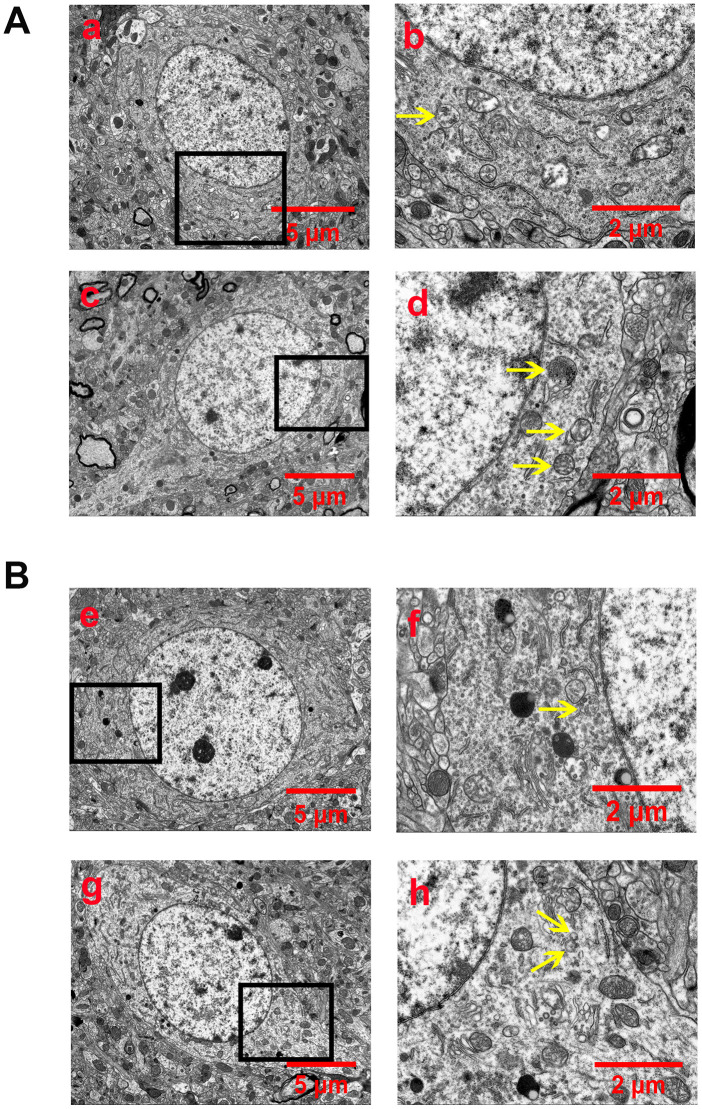
**EA pretreatment inhibited the formation of autophagosomes and autophagolysosomes (yellow arrow) in cortical neurons of rats with CIR injury.** (**A**) Autophagosomes and autophagolysosomes in neurons of the peri-ischemic cortex in the sham group (a and b) and the I/R group (c and d). (**B**) Autophagosomes and autophagolysosomes in neurons of the peri-ischemic cortex in the I/R + EA group (e and f) and the I/R + EA + EX527 group (g and h). The images of b, d, f, and h indicated the enlarged area of the squares in a, c, e, and g, respectively. Scale bar: a, c, e, g, 5 μm; b, d, f, h, 2 μm.

Autophagosomes or autophagolysosomes are observed by electron microscopy and have been considered the gold standard for identifying autophagy. Thus, we detected the formation of autophagosomes in neurons of the peri-ischemic cortex by electron microscopy. As shown in Figure 3, neurons in the rat of the sham group were relatively normal and showed a comparative normal-looking nucleus, mitochondria, endoplasmic reticulum, and ribosomes. After 24 h of reperfusion, increased autophagosomes and autophagolysosomes could be found in the neurons. The cellular morphology was heavily impacted. The mitochondria were abnormally swollen and lost their integrity. Some mitochondrial cristae had broken, decomposed, and even disappeared. The EA pretreatment decreased the number of autophagosomes and autophagolysosomes, as well as alleviated the injury of the ultrastructure of neurons. In addition, the morphology of the mitochondria was relatively normal, and the nucleus and membrane of neurons were clearly visible. However, EX527 abrogated the beneficial effect of EA on the ultrastructure of neurons and increased the number of autophagosomes and autophagolysosomes.

### EA pretreatment reduced the expression of LC3 while increased the expression of p62 in cortical neurons

**Figure 4:**
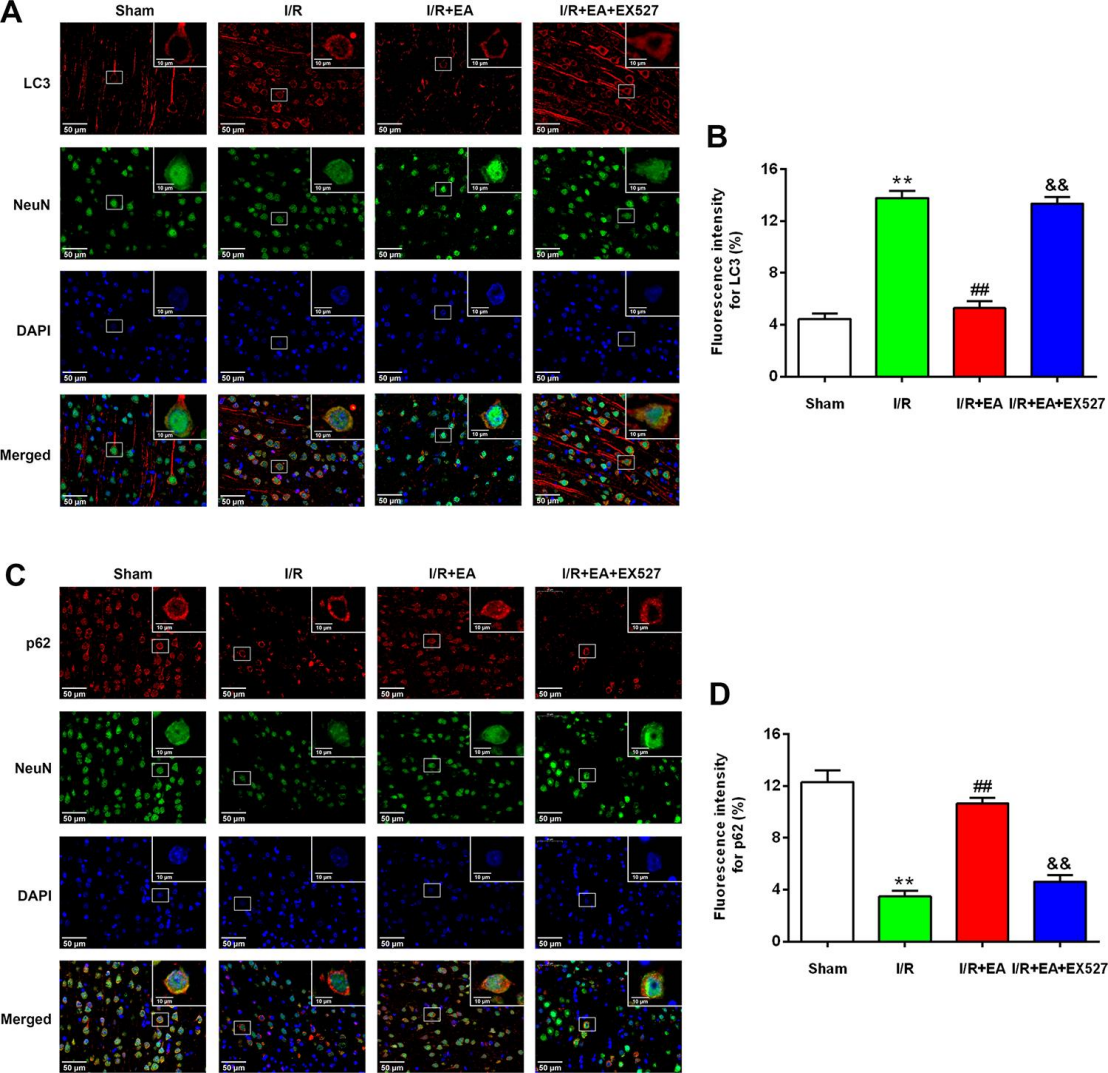
**EA pretreatment inhibited the expression of LC3 in NeuN-positive neurons of the peri-ischemic cortex, while promoted the expression of p62 in NeuN-positive neurons of the peri-ischemic cortex after 24 h of reperfusion.** (**A**) Representative images of LC3 (red)/NeuN (green) double-labeled staining (400×). High magnification images are shown in the small windows (1000×). Scale bar, 400×: 50 μm; 1000×: 10 μm. (**B**) Mean fluorescence intensity for LC3 in neurons of the peri-ischemic cortex. (**C**) Representative images of p62 (red)/NeuN (green) double-labeled staining (400×). High magnification images are shown in the small windows (1000×). Scale bar, 400×: 50 μm; 1000×: 10 μm. (**D**) Mean fluorescence intensity for p62 in neurons of the peri-ischemic cortex. Data were presented as the mean ±SEM (*n*=3). ^**^P<0.01 *vs*. sham group. ^##^*P*<0.01 *vs*. I/R group; ^&&^*P*<0.01 *vs*. I/R + EA group.

After 24 h of reperfusion, we examined the activity and location of autophagy by immunofluorescent staining. Since LC3 and p62 were both used as specific markers of autophagy, we evaluated LC3/NeuN and p62/NeuN in each group. The immunofluorescence results revealed that LC3 and p62 mainly existed in cortical neurons (Figure 4A, 4C). Compared with the sham group, the level of LC3 was dramatically increased in the I/R group while the expression of p62 was significantly decreased in cortical neurons. The EA pretreatment significantly downregulated the expression level of LC3, upregulated the expression of p62 in cortical neurons. However, pretreatment with EX527 blocked the decrease of LC3 and the increase of p62 in cortical neurons (Figure 4B, 4D).

### EA pretreatment decreased the ratio of LC3-II/LC3-I and upregulated the protein expression level of p62 after CIR injury

**Figure 5:**
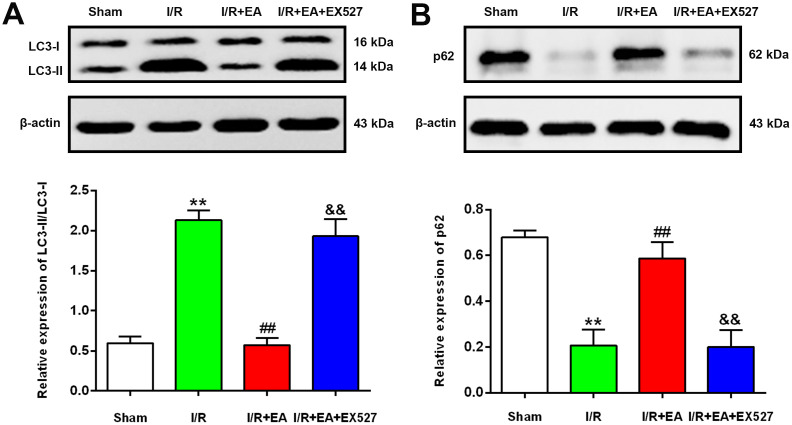
**EA pretreatment down-regulated the protein level of LC3, while up-regulated the p62 level in the peri-ischemic cortex after 24 h of reperfusion.** (**A**) Protein band of LC3 and the ratio of LC3-II/LC3-I. (**B**) Protein band and relative protein level of p62 in the peri-ischemic cortex. β-actin was used as a loading control. Data were presented as the mean ±SEM. (*n*=3). ^**^P<0.01 *vs*. sham group. ^##^*P*<0.01 *vs*. I/R group; ^&&^*P*<0.01 *vs*. I/R + EA group.

To further examine the effect of EA pretreatment at the GV20, LI11 and ST36 acupoints on autophagy in the peri-ischemic cortex, the protein levels of LC3 and p62 were evaluated by western blot after 24 h of reperfusion. The result showed that the ratio of LC3-II/LC3-I was significantly increased, while the protein expression of p62 was markedly decreased in the I/R group versus the sham group. In comparison to the I/R group, the LC3-II/LC3-I ratio was significantly decreased, and the protein level of p62 was significantly increased in the I/R + EA group. However, the I/R + EA + EX527 group showed an increase in the ratio of LC3-II/LC3-I and a decrease in the level of p62 compared with the I/R + EA group (Figure 5).

### EA pretreatment increased the protein level of SIRT1, contributed to the deacetylation of FOXO1, and decreased the protein level of Atg7 after CIR injury

**Figure 6:**
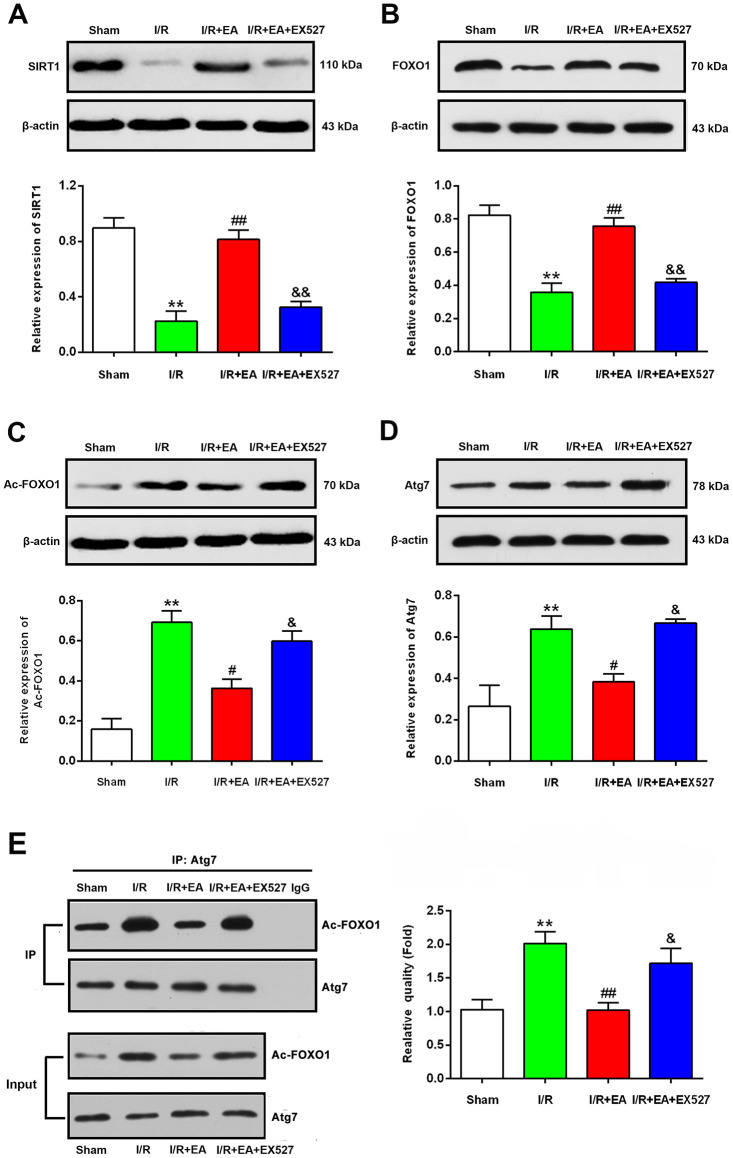
**EA pretreatment up-regulated the protein levels of SIRT1, FOXO1, down-regulated the protein levels of Ac-FOXO1 and Atg7 in the peri-ischemic cortex after 24 h of reperfusion.** (**A**) Protein band and relative expression of SIRT1 in the peri-ischemic cortex. (**B**) Protein band and relative expression of FOXO1 in the peri-ischemic cortex. (**C**) Protein band and relative expression of Ac-FOXO1 in the peri-ischemic cortex. (**D**) Protein band and relative expression of Atg7 in the peri-ischemic cortex. (**E**) Co-IP between Atg7 and Ac-FOXO1 in the peri-ischemic cortex and the relative quality of Ac-FOXO1 after normalization with Atg7, and input results of Atg7 and Ac-FOXO1. β-actin was used as a loading control. Data were presented as the mean ± SEM (*n*=3). ^**^P<0.01 *vs*. sham group. ^#^*P*<0.05 and ^##^*P*<0.01 *vs*. I/R group; ^&^*P*<0.05 and ^&&^*P*<0.01 *vs*. I/R + EA group.

To explore the underlying mechanisms by which pretreatment with EA at the GV20, LI11, and ST36 acupoints may inhibit autophagy *via* the SIRT1-FOXO1 signaling pathway during CIR injury, we evaluated the protein expression levels of SIRT1, FOXO1, Ac-FOXO1 and Atg7 at 24 h after reperfusion. The western blot results showed that the protein levels of SIRT1 and FOXO1 were significantly decreased in the I/R group compared with the sham group, while the Ac-FOXO1 and Atg7 levels were significantly increased. The EA pretreatment markedly increased the protein expression of SIRT1 and FOXO1 and decreased the levels of Ac-FOXO1 and Atg7 after 24 h of reperfusion. However, the EX527 administration abrogated the increase of SIRT1 and FOXO1 levels and increased the levels of Ac-FOXO1 and Atg7 (Figure 6).

### EA pretreatment suppressed the interaction of Ac-FOXO1 and Atg7 after CIR injury

We further examined the effect of EA pretreatment on the interaction of Ac-FOXO1 and Atg7 in the peri- ischemic cortex after 24 h of reperfusion. The results of the co-immunoprecipitation assay revealed that the interaction of Ac-FOXO1 and Atg7 was dramatically increased in the I/R group compared with the sham group. The EA pretreatment significantly suppressed the interaction of Ac-FOXO1 and Atg7 compared with the I/R group. However, the EX527 administration abrogated the suppression on the interaction of Ac-FOXO1 and Atg7 induced by the EA pretreatment (Figure 6E).

## DISCUSSION

In the current study, we investigated the neuroprotective effects of EA pretreatment at the GV20, LI11, and ST36 acupoints against CIR injury and the underlying mechanisms of modulating autophagy. The present data showed that pretreatment with EA at the GV20, LI11, and ST36 acupoints continuously for 5 days dramatically reduced the volume of cerebral infarction, alleviated neurological deficits, and increased the dendritic spine density of neurons in the peri-ischemic cortex. In addition, EA pretreatment markedly reduced the expression of LC3 in neurons and increased the expression of p62 in neurons of the cerebral cortex. Western blotting also showed that the ratio of LC3-II/LC3-I decreased and the level of p62 increased after the EA pretreatment. Additionally, EA pretreatment further enhanced the expression of SIRT1, inhibited the level of Ac-FOXO1 and Atg7, and suppressed the interaction of Ac-FOXO1 and Atg7. However, all those effects of EA pretreatment were abrogated by the SIRT1 inhibitor EX527. All these findings innovatively indicate that EA pretreatment exhibits a beneficial efficacy against focal CIR injury by suppressing autophagy through activating the SIRT1-FOXO1 signaling pathway.

Acupuncture, a distinctive complementary therapy, has been used to prevent and treat various diseases for thousands of years in China and other Southeast Asian countries [36–38]. As early as 1979, acupuncture needles have been approved by the FDA as Class III (investigational) medical device and allowed their clinical use by licensed practitioners [39]. In the same year, 43 kinds of diseases and disorders that can be cured by acupuncture, such as nausea, vomiting, pain, as well as ischemic stroke, were approved by the World Health Organization. EA, a novel therapy based on traditional acupuncture combined with modern electric stimulation to the acupoints, has a nearly 200-year history which was the first applied by French physicians, Louis Berlioz, in his clinical work in the early eighteenth century [40]. At present, EA has been generally acknowledged worldwide due to the remarkable neuroprotective effect in treating ischemic cerebrovascular disease such as ischemic stroke [41, 42]. After reviewing the references regarding acupuncture treatment for ischemic cerebrovascular diseases, we found that GV20, LI11, and ST36 were the most frequently selected acupoints [23, 42]. According to the meridian theory of traditional Chinese medicine, “*Baihui*” (GV20) is one of the most important acupoints of *Du* Meridian (the government vessel) and is commonly used in neurology and psychiatry [43]. “*Quchi*” (LI11) and “*Zusanli*” (ST36) are sea points of the *Yangming* large intestine meridian of the hand and *Yangming* stomach meridian of the foot, and they are the most commonly used to clinically treat stroke in China [44]. Hence, in this study, we screened out the three acupoints to investigate the efficacy of EA pretreatment in rats with CIR injury.

For decades, numerous clinical trials have evaluated the efficacy and safety of EA treatment for ischemic cerebrovascular diseases especially ischemic stroke as a complementary and alternative therapy [18, 45]. A randomized controlled trial suggested that EA at the LI11 and ST36 acupoints was beneficial and safe for the treatment of post-stroke spasticity such as muscle tone, motor function, and activities of daily living [46]. Another clinical study showed that motor functions of the limbs and the activities of daily living in hemiplegic patients caused by acute cerebral infarction were improved significantly after EA treatment at GV20, LI11, and ST36 and associated with the reduction of serum neuron-specific enolase, soluble protein-100B and endothelin levels [47]. Moreover, clinical research also indicated that EA at GV20 might activate the cerebral motor areas bilaterally and induce the excitation of nerve tissue related to motion, thus further contributing to the recovery of motor function [48]. However, the underlying mechanisms responsible for EA treatment efficacy for ischemic stroke remain poorly understood.

CIR injury is an aggravating secondary injury during thrombolytic therapy of ischemic cerebrovascular disease. Accumulating experimental researches have demonstrated that the superior functions induced by EA in treating CIR injury include effectively restoring blood supply, reducing the cerebral infarct volume, and improving learning and memory impairment [49–52]. These beneficial effects of EA pretreatment have been confirmed to be achieved by benignly regulating a series of pathological reactions. Zou et al [20] reported that EA pretreatment at GV20 dramatically reduced the blood-brain barrier permeability and brain edema through suppressing the degradation of tight junction proteins and inhibiting the expression of p-caveolin-1 in endothelial cells, thereby attenuating CIR injury in rats. Liu et al [53] found that EA treatment at LI11 and ST36 acupoints in rats with MCAO decreased the expression of inflammatory cytokines including tumor necrosis factor-alpha, interleukin-1 beta, and interleukin-6, reduced the volume of cerebral infarction, and improved nerve motor function, suggesting that the neuroprotection afforded by EA against CIR injury involved an anti-inflammation effect. Studies have suggested that EA pretreatment at GV20 [24] and LI11 and ST36 acupoints [54] exerts neuroprotective effects following CIR by suppressing autophagy. Pretreatment with EA at GV20 could significantly decrease the ratio of LC3-II/LC3-I and increase the expression levels of p-AKT and p-mTOR in rats with CIR injury, suggesting that EA may inhibit autophagy through activating the AKT-mTOR pathway [30]. Another similar study showed that EA at LI11 and ST36 acupoints decreased the levels of LC3-II/LC3-I, Atg13, ULK1 and Beclin 1, and increased the expression of mTORC1, indicating that the potential mechanisms underlying autophagy inhibition by EA might be mediated by the activation of the mTORC1-ULK complex-Beclin1 pathway [55]. However, the underlying mechanisms of EA pretreatment at GV20, LI11, and ST36 have not been completely elucidated. We herein tried to reveal the possible underlying mechanism of EA pretreatment at GV20, LI11, and ST36 in rats with CIR injury.

In the present study, we found that EA at the GV20, LI11, and ST36 acupoints effectively reduced the cerebral infarct volume and improved neurological impairment symptoms in rats with CIR injury, indicating that EA pretreatment exerted a beneficial effect in rats with CIR injury. Dendritic spines are small, thin, specialized protrusions from neuronal dendrites and consist of a dense network of cytoskeletal, transmembrane molecules, and numerous surface receptors [56]. Dendritic spines are sensitive to pathological changes in the brain, and they are critical in cognitive and motor function, as well as memory formation. In our study, we found that the dendritic spine density was reduced in the cortical neurons of rats with CIR injury; however, EA pretreatment markedly increased dendritic spine density in the cortex, suggesting that EA pretreatment at GV20, LI11, and ST36 contributed to the restoration of synaptic-dendritic plasticity and motor function. Similar results were presented in previous studies. Lin et al [57] found that the number and density of dendritic spine were decreased in the hippocampus of rats with CIR injury and EA treatment at GV20 and “*Shenting*” (GV24) was shown to significantly increase the dendritic spine density in the hippocampus. Liu et al [58] also reported that the density of dendritic spines and the number of synapses in the hippocampal CA1 pyramidal cells were significantly increased on the 14^th^ day after MCAO surgery when given treatment with EA at the acupoints of GV20 and GV24.

Autophagy is a cellular degradation and recycling process that is highly conserved in all eukaryotes. Animal experimental studies have demonstrated that autophagy plays a double-edged sword role in the central nervous system following CIR injury, especially in neurons [13, 59–61]. During the first few hours of reperfusion, autophagy played a protective role in CIR injury through degrading damaged organelles and misfolded proteins with the lysosomal system [61–64]. At later stages of reperfusion, excessive autophagy would over-degrade the organelles and proteins with normal functions, finally leading to autophagic cell death and secondary damage of cells and tissues [24, 55, 65]. Published data have shown that CIR induces autophagosome formation and autophagy through activating the autophagy-related proteins, LC3 [13, 29]. Autophagosome is a double-membrane vesicle containing damaged organelles or proteins. After fusion with the lysosome, autophagosome evolves into autophagolysosome, a vesicle with a monolayer membrane [66]. The present study showed that pretreatment with EA decreased the number of autophagosomes and autophagolysosomes, indicating that EA pretreatment has a repressive effect on the regulation of autophagosomes after CIR injury. LC3, one of the most important participants in the occurrence of autophagy, plays a positive role both in the formation of autophagosomes and the degradation of autophagolysosomes. It has been confirmed that LC3 exists in two forms, LC3-I (soluble form) and LC3-II (membrane-bound form), and LC3-II is enriched on the vesicle membrane to promote the formation of mature autophagosomes [67]. Moreover, LC3-II is also involved in the degradation of autophagy by binding to the C terminal of p62 [68]. Our study revealed that EA pretreatment at GV20, LI11, and ST36 decreased the ratio of LC3-II/LC3-I and the expression of LC3 in NeuN-positive neurons in the peri-ischemic cortex and increased the protein level of p62 and the expression of p62 in NeuN-positive neurons in the peri-ischemic cortex, suggesting that the neuroprotective effect of EA is related to the inhibition of neuronal autophagy. Our findings were also confirmed by other research, Wu et al [24] found that the number of autophagosomes and the expression of the LC3-II were significantly increased after 12 h of I/R, while EA pretreatment at GV20 decreased the expression of LC3-II and the number of autophagosomes in the ischemic cortex. Another study also showed that EA at the LI11 and ST36 acupoints decreased the level of LC3-II/LC3-I in the peri-ischemic cortex [55]. In addition, Li et al [69] reported that EA at the “*Shuigou*” (GV26) acupoint significantly increased the expression of p62 after 24 h of reperfusion. All these studies suggested that autophagy may be a potential therapeutic target for the treatment of ischemic cerebrovascular disease by utilizing EA.

Previous studies have indicated that autophagy was involved in the alterations of neuronal dendritic morphology and that Atg7 and Atg5 might play an indispensable role in the maintenance of axonal homeostasis and the prevention of axonal degeneration [70–72]. Moreover, Ruan et al [73] found that autophagy occurred in both phagocytizing damaged organelles and phagocytizing synaptic structures after ischemia, while inhibition of autophagy in dendrites and spines after ischemia might contribute to the survival of pyramidal neuron in the CA1 area. In the present study, we found that the activation of autophagy led to a decrease in dendrite spine density in rats with CIR injury, while EA pretreatment inhibited autophagy and increased the density of dendritic spines, Thus, neuronal autophagy might result in a decrease in dendrite spine density while EA pretreatment increases the density of dendritic spine by suppressing neuronal autophagy.

SIRT1, a conserved NAD-dependent histone deacetylase, has been shown to promote dendrite outgrowth and axon development in embryonic hippocampal neurons [74]. Accumulating evidence also demonstrated that SIRT1 played a crucial role in modulating apoptosis and autophagy for its NAD-dependent deacetylase activity [34, 75, 76]. As a downstream target of SIRT1, FOXO1 has been confirmed to be deacetylated by SIRT1 [34, 75, 76]. It has also been reported that FOXO1 plays a crucial role in controlling the development of connectivity of DG neurons, maintenance of autophagic degradation activity, and morphological maturation of developing neurons [78, 79]. Studies have indicated that SIRT1 could inhibit inflammation and apoptosis to attenuate CIR injury by deacetylating FOXO1 [80, 81]. A recent study reported that EA pretreatment could significantly increase the expression level of SIRT1 in rats with CIR injury [82]. In this study, we innovatively found that the protein expression of SIRT1 was remarkably downregulated, while the Ac-FOXO1 level was dramatically upregulated following 24 h of reperfusion, which could be reversed by EA pretreatment, suggesting that EA pretreatment promoted the regeneration of neuronal dendrites *via* upregulating the expression of SIRT1 and FOXO1. A similar result has also been reported in another disease. Yang et al [83] indicated that EA treatment could markedly increase the protein level of SIRT1 and FOXO1 in obese rats. Additionally, substantial evidence suggested that in response to stress, acetylated FOXO1 (Ac-FOXO1) binds to Atg7, an E1-like protein, to induce autophagy leading to cell death [35, 84]. Our results provided evidence that the interaction of Ac-FOXO1 and Atg7 was related to the induction of autophagy in rats with CIR injury. Data of the co-immunoprecipitation assay confirmed that the interaction of Ac-FOXO1 and Atg7 was significantly increased in the peri-ischemic cortex in rats with CIR injury, while EA pretreatment at GV20, LI11, and ST36 significantly abolished this upregulation. In contrast, EX527 blocked the deacetylation of FOXO1 and increased the interaction of Ac-FOXO1 and Atg7. The above results indicated that the activation of SIRT1 might mediate the neuroprotective effects of EA through de-acetylating FOXO1 and reducing the interaction of Ac-FOXO1 and Atg7, thus attenuating autophagy of the ischemic brain.

## CONCLUSION

**Figure 7:**
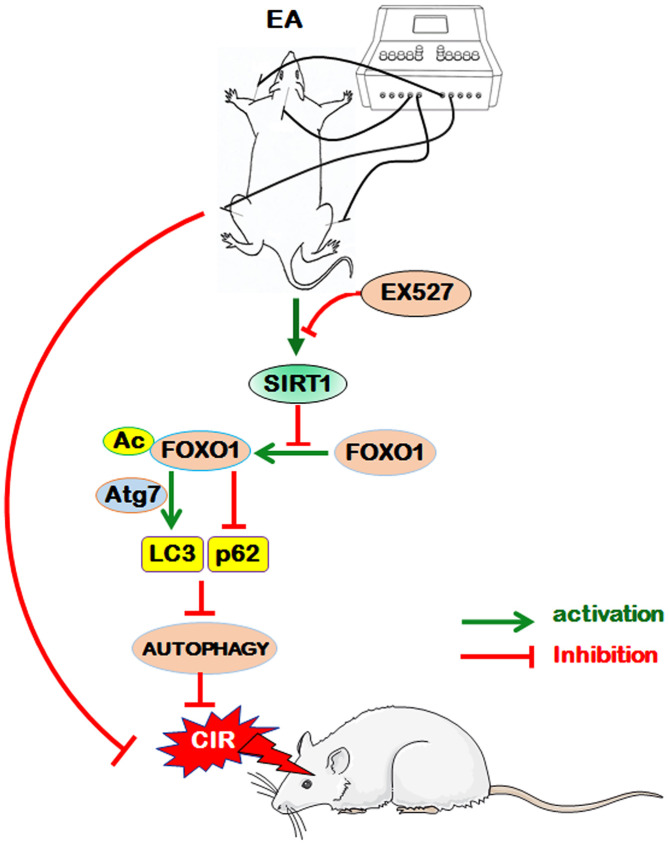
**The proposed scheme for the possible mechanism of EA pretreatment suppresses CIR-induced autophagy *via* the SIRT1-FOXO1 signaling pathway.** EA pretreatment at GV20, LI11, and ST36 significantly alleviated neurological deficits, reduced infarct volume, increased the dendritic spine density of cortical neurons, decreased the LC3-II/LC3-I ratio, and up-regulated p62 level. The above beneficial effects may be achieved by the activation of SITR1 and the inhibition of acetylation of FOXO1, as well as the suppression of Atg7 expression and the interaction of Ac-FOXO1 and Atg7.

EA pretreatment at the GV20, LI11, and ST36 acupoints effectively ameliorated neurological deficits and cerebral infarct volume, markedly increased the density of dendritic spines and suppressed autophagy in ischemic cortical neurons 24 h after reperfusion, indicating that EA pretreatment exerted a neuroprotective effect in the reperfusion phase after MCAO in rats. Further analysis revealed that EA pretreatment protected the brain tissue against CIR-induced autophagy *via* the SIRT1-FOXO1 signaling pathway (Figure 7). However, whether EA inhibits autophagy through regulating other signing pathways and whether EA stimulation at other acupoints can also modulate autophagy through the same pathway remain to be investigated, and whether other independent risk factors for ischemic stroke such as ALDH2, which has a close association with stroke [85], would be possibly involved in the efficacy of EA pretreatment also need to be further studied. In brief, we presumed that autophagy may be a potential therapeutic target for ischemic cerebrovascular diseases by EA pretreatment in the present study, and we will investigate unresolved issues in further studies to provide scientific theoretical perspectives for EA pretreatment against ischemic stroke.

## MATERIALS AND METHODS

### Animals and groups

A total of 56 male Sprague–Dawley rats (220 ± 20 g) were purchased from the Laboratory Animal Center of China Tree Gorges University (Yichang, China; license No. SCXK (E) 2017-0012). The rats were randomly and evenly divided into 4 groups (*n*=14): the sham-operated control group (sham group); the MCAO control group (I/R group); the MCAO + EA pretreatment group (I/R + EA group) and the MCAO + EA pretreatment + EX527 group (I/R + EA + EX527 group). All rats were maintained under a 12-12-hour light-dark cycle environment. The room temperature was controlled at (22 ± 3) °C, with (60 ± 5) % humidity, and adequate food and water were provided. The animal experiments were approved by the Laboratory Animal Ethical Committee of Three Gorges University, China (No. 20180904C, approved at 2018-9-11).

### EA pretreatment

Rats in the I/R + EA group and the I/R + EA + EX527 group received EA treatment. Stainless acupuncture needles (diameter 0.35 mm and length 25 mm, Wuxi Jiajian Medical Instrument Co., Ltd, Wuxi, China) were inserted 2-3 mm deep into “*Baihui*” (GV20, located in the intersection of the sagittal midline and the line between the two ears), “*Quchi*” (LI11, located in the depression lateral to the anterior aspect of the radial proximal elbow) and “*Zusanli*” (ST36, located in the posterolateral side of the knee joint, approximately 5 mm below the capitula fibula) acupoints on the left limb. Animals were anesthetized and stimulated by an EA instrument (G6805-2A, Shanghai Huayi Medical Instrument Factory, Shanghai, China). The stimulation parameters were set as dense disperse waves of 2/15 Hz (adjusted to the muscle twitch threshold) and an intensity of 1 mA for 30 min/day, once per day for 5 consecutive days prior to MCAO (Figure 1A and 1B).

### Drug administration

As reported previously [80, 81], the SIRT1 inhibitor EX527 (NO.1541, Selleck Chemicals, Houston, TX, USA) was first dissolved in dimethyl sulfoxide and diluted to a final concentration with normal saline (final dimethyl sulfoxide concentration <2%). The I/R + EA + EX527 group rats received EA treatment once daily for 5 days and received the subarachnoid administration of the SIRT1 inhibitor EX527 at a dose of 5 mg/kg every 2 days, four times in total before MCAO.

### Establishment of CIR injury model in rat

The rat model of MCAO was established as previously described in the study by Longa et al [86]. Briefly, 30 mins after the last EA pretreatment, the rats were anesthetized with 10% chloral hydrate (350 mg/kg) by intraperitoneal injection. The right common carotid artery (CCA), external carotid artery (ECA), and internal carotid artery (ICA) were exposed. A monofilament nylon suture was inserted from the right CCA to the ICA, approximately 18-20 mm towards the circle of Willis to block the origin of the right middle cerebral artery. After 90 min of MCAO-induced cerebral ischemia, the suture was slowly pulled out to allow reperfusion for 24 h. The rectal temperature of the rats was maintained at 37 °C throughout the surgical procedures. The rats were placed in an environment at room temperature and resumed a normal diet after surgery. In the sham group, only the artery was isolated and no embolus was introduced.

### Assessment of neurological scores

As previously described in the study by Longa et al [86], the neurological scores were assessed after 24 h of reperfusion following 90 min of ischemia. The score given to each rat at the completion of the evaluation and the scoring criteria were as follows: 0 = no neurological deficit; 1 = the left forelimbs unable to fully extend when held by tail; 2 = circling to the contralateral side and not able to go straight; 3 = difficult to walk and the body was slumped to the left when walking; 4 = unable to walk spontaneously along with a possible loss of consciousness. Scores between 1 and 3 indicated rats have undergone successful induction of focal cerebral ischemia/reperfusion, and rats with these scores were accepted.

### Measurement of the infarct volume

After 24 h of reperfusion, the infarction volume was assessed by 2,3,5-triphenyl tetrazolium chloride (TTC, Sigma-Aldrich St. Louis, MO, USA) staining. Briefly, at the end of the neurological examination, the rat brains were quickly dissected and then placed in a freezer at -20 °C for 20 min. Each brain was sliced into five 2-mm-thick sections and immediately immersed in 0.2% TTC for 30 min at 37 °C in the dark. Then, the sections were placed in a 4% paraformaldehyde solution for 24 h. The TTC-stained sections were photographed using a high-resolution digital camera, and the infarct area was determined with an Image-pro plus 6.0 (Media Cybernetics, Maryland, MD, USA). The total infarct volume was analyzed using 5 slices from each brain and calculated by the following formula: infarct volume (%) = (infarct volume/total volume) ×100. The percentage of infarct volume was subsequently used for statistical analysis.

### Golgi-Cox staining

Three rats were randomly selected from each group. The rat brains were rapidly exfoliated and then fixed in 4% paraformaldehyde solution for 24 h. The brains were gently rinsed 3 times with phosphate-buffered saline (PBS, pH 7.4) and then completely immersed in the Rapid Golgi Stain Kit (G1069, Servicebio, Wuhan, China) for 14 days. The brains were dehydrated with a 15% sucrose solution for 24 h at 4 °C and a 30% sucrose solution for 2 days. The brains were washed with double-distilled water for 1 min, immersed in concentrated ammonia for 30 min, fixed with acid-hardened fixer bath for 30 min, and finally dehydrated using a 30% sucrose solution for 2 days at 4 °C. A series of 100 μm thick sections were sliced from the peri-ischemic frozen cortex tissue, and the slices were sealed with glycerin gelatin. The dendritic spine density was analyzed for pyramidal neurons within the peri-ischemic area of cortex. For each cell, at least 50 μm long segments of terminal basilar densities and apical densities were obtained. The same cells were traced at 1000× magnification. The spine number was counted, and the exact length of the dendritic segment was calculated to yield spines at 10 μm.

### Transmission electron microscopic examination

After 24 h of reperfusion, the rats were anesthetized and then perfused transcardially with a physiological saline solution followed by 0.1 M PBS containing 4% paraformaldehyde and 0.25% glutaraldehyde. The peri-ischemic cortical tissue was cut into blocks of 1 mm × 1 mm × 1 mm and post-fixed with 2.5% glutaraldehyde at 4 °C. The pieces were washed 3 times with PBS and then processed by post-fixation with 1% osmium tetroxide in PBS for 2 h at 4 °C. The tissue blocks were then washed 3 times with PBS, dehydrated in graded ethanol and then embedded in epoxy resin, which was followed by polymerization for 24 h at -80 °C. The blocks were sectioned by a Leica ultramicrotome (Leica, Wetzlar, Germany) into ultrathin sections (60-70 nm). The sections were post-stained with 3% lead citrate and visualized using a transmission electron microscope (Hitachi, Tokyo, Japan) to observe the structure and number of mitochondria, autophagosomes, autolysosomes, and lysosomes.

### Immunofluorescence staining

After 24 h of reperfusion, the rats were anesthetized and perfused transcardially with a physiological saline solution followed by 4% paraformaldehyde in PBS. The brains were rapidly removed and post-fixed with 4% paraformaldehyde overnight at 4 °C. Each brain tissue was sliced into 20 μm coronal sections, preincubated in 0.3% Triton X-100 in PBS for 30 min, and then incubated with primary antibodies against LC3B (1:200, Proteintech Group, Wuhan, China) and NeuN (1:1000, Abcam, Cambridge, UK) or p62 (1:200, Proteintech Group) and NeuN overnight at 4 °C. After rinsing 3 times with PBS, the sections were incubated with fluorescent (Cy3)-labeled goat anti-rabbit IgG and fluorescent (FIFC)-labeled goat anti-mouse IgG (1:200, Servicebio) for 2 h at room temperature. The nuclei were counterstained with 4′,6-diamidino-2-phenylindole (DAPI) for 5 min without light. The sections were mounted on a coverslip with an anti-fluorescence quenching mounting medium (Beyotime Biotechnology, Shanghai, China). All sections were observed with a fluorescence microscope (Leica, Wetzlar, Germany) by an investigator who did not know the experimental design. The fluorescence intensity of positive cells in three non-overlapping fields (400×) was calculated using ImageJ software.

### Western blot analysis

Twenty-four hours after reperfusion, the total proteins were extracted from the peri-ischemic cortical tissue supplied by the right MCAO, the corresponding area in the sham rats were homogenized, and the total proteins were extracted with a lysis buffer. The protein concentrations were determined using a spectrophotometer. The western blot analysis was separated by 10-12% sodium dodecyl sulfate-polyacrylamide gel electrophoresis (SDS-PAGE). Equal amounts of total protein (30 μg) from each sample were loaded onto the gels for the western blot analysis. Following electrophoresis, proteins were transferred onto polyvinylidene fluoride (PVDF) membranes (Millipore, MA, USA) for 1.5 h. The membranes subsequently were blocked with 5% skim milk for 1 h at room temperature and then incubated with specific polyclonal antibodies against LC3B (1:500, Proteintech Group), p62 (1:2000, Proteintech Group), FOXO1 (1:1000, Proteintech Group), SIRT1 (1:1000, Cell Signaling Technology, MA, USA), Atg7 (1:1000, Cell Signaling Technology), Ac-FOXO1 (1:500, Affinity Biosciences, OH, USA) and β-actin (1:2000, Servicebio) at 4°C overnight. The PVDF membranes were washed 3 times (10 min every time) in Tris-buffered saline/Tween-20 (TBST) and then incubated with horseradish peroxidase-conjugated goat anti-rabbit secondary antibody (1:3000, Servicebio) at the room temperature for 1 h. The PVDF membranes were washed 3 times in TBST. Immunoreactivity was detected using enhanced chemiluminescent autoradiography (Bioroc Pharmaceutical and Biotech Co., Ltd, Tianjin, China) in accordance with the manufacturer's instructions. β-actin was used as the loading control. The analysis of western blot was performed using ImageJ software.

### Co-immunoprecipitation assay

Twenty-four hours after reperfusion, the peri-ischemic cortical tissues were lysed in 1 mL cold IP lysis buffer (G2038, Servicebio) containing a complete protease inhibitor cocktail (G2006, Servicebio). The brain tissue lysates were used for immunoprecipitation with anti-Atg7 (1:100, Cell Signaling Technology). Subsequently, 2 to 4 μg of antibody was added to 1 mL of brain tissue lysate and incubated at 4 °C overnight. After the addition of Protein A/G Plus Agarose beads (IP05, Millipore), the incubation was continued for 1 h. Immunoprecipitants were extensively washed with lysis buffer, eluted with SDS loading buffer (G2031, Servicebio) by boiling for 5 min, and then processed for immunoblotting with the indicated antibodies of Ac-FOXO1 (1:500, Affinity Biosciences). Immunoreactivity was detected using enhanced chemiluminescent autoradiography (Bioroc Pharmaceutical and Biotech Co., Ltd, Tianjin, China) in accordance with the manufacturer's instructions. The analysis of immunoblotting was performed using ImageJ software.

### Statistical analysis

Data were analyzed using SPSS19.0 statistical software and presented as the mean ± standard error of the mean (SEM). Statistical significance was analyzed by one-way analysis of variance (ANOVA) with Tukey’s or Dunnett’s post-test. Values of *P*<0.05 were considered to indicate statistical significance.
